# Economic Burden of Congenital Cytomegalovirus Disease on Families in the United States

**DOI:** 10.3390/v18090973

**Published:** 2026-09-03

**Authors:** Philip O. Buck, Carolyn Sweeney, Mihaela Georgieva, Colin Kunzweiler, Harout Tossonian, Kimberly Boyle, Costel Chirila, Rebecca Crawford, Sorrel Wolowacz, Megan H. Pesch

**Affiliations:** 1 ModernaTx, Inc., Cambridge, MA 02142, USA; pobuck@yahoo.com (P.O.B.); mihaela.v.georgieva@gmail.com (M.G.); harout.tossonian@modernatx.com (H.T.); 2 RTI Health Solutions, Durham, NC 27713, USA; csweeney@rti.org (C.S.); kboyle@rti.org (K.B.); cchirila@rti.org (C.C.); 3 RTI Health Solutions, Manchester M20 2LS, UK; rcrawford@rti.org (R.C.); swolowacz@rti.org (S.W.); 4 Division of Developmental and Behavioral Pediatrics, Department of Pediatrics, University of Michigan, Ann Arbor, MI 48109, USA; megan.pesch@gmail.com

**Keywords:** congenital cytomegalovirus, family spillover effects, economic burden, health-state utilities, survey

## Abstract

**Objective:** The objective of this study was to assess the economic family spillover effects of childhood congenital cytomegalovirus (cCMV) in the US. **Methods:** This cross-sectional, Web-based study collected economic and health-state utility data between August 2024 and April 2025 using three surveys targeting caregivers of children with cCMV, children with cCMV, and their siblings. **Results:** Respondents included 227 caregivers, 211 children with cCMV, and 173 siblings. A high economic burden on families of children with cCMV was reported, including total mean annual costs (out-of-pocket medical and personal care expenses and work- or productivity-related costs) of $83,140 and lifetime out-of-pocket home adaptation/specialized schooling costs of $4713. The mean caregiver EQ-5D-5L utility score was 0.86, which is comparable to US general-population norms. Mean Health Utilities Indexes Mark 2 and 3 scores varied within the ranges of 0.38–0.83 and 0.03–0.73, respectively, among children with cCMV. Adult siblings reported a mean EQ-5D-5L utility of 0.90. Siblings aged 4–7 and 8–17 years reported mean EQ-5D-Y-3L utilities of 0.90 and 0.92, respectively. **Conclusions:** This is the first study to report the family spillover effects of caring for children with cCMV in the US. The results confirm a considerable, multifaceted burden experienced by children with cCMV and their families, spanning health-state utility and economic strains.

## 1. Introduction

In the United States (US), congenital cytomegalovirus (cCMV) is the leading infectious cause of birth defects, estimated to affect 4.5 to 5.0 per 1,000 (i.e., 1 in 200 to 1 in 220) live births [1,2,3]. cCMV occurs when infection with cytomegalovirus (CMV), a common human viral pathogen of the Herpesviridae family, is transmitted through the placenta to a growing fetus [4]. Maternal CMV infection can result in adverse prebirth impacts (e.g., miscarriage), and babies born with CMV can experience life-long impacts, including sensorineural hearing loss (SNHL), developmental delays, cerebral palsy, epilepsy, and intellectual disability [4,5,6,7]. These sequelae have been linked to worse school performance and lower health-related quality of life in children with cCMV than in children without cCMV [7].

The severity of symptoms associated with cCMV at birth can be categorized as asymptomatic (no signs or symptoms), asymptomatic with isolated SNHL (no signs or symptoms except SNHL), mildly symptomatic (transient signs), and moderately to severely symptomatic (signs of end-organ damage including central nervous system [CNS] involvement) [8]. The presence of CNS involvement (e.g., microcephaly or radiographic abnormalities on brain imaging) is a predictor of long-term adverse health outcomes (i.e., epilepsy and/or cerebral palsy) [9]. However, all children with cCMV remain at heightened risk for additional sequelae, such as progressive SNHL, autism, or vestibular dysfunction, during childhood and adolescence [10,11,12,13].

Families can also experience impacts of caring for a child with cCMV, termed family spillover effects, which can include out-of-pocket costs (e.g., transportation, education, and specialized housing), work/productivity impairment, and impacts on health status [7,14,15]. In a United Kingdom (UK) study, the health status of parents of children with cCMV with parent-reported severe health effects was numerically lower versus parents of children with less severe effects [16]. In the same study, the parent-reported quality-adjusted life years of children with severe effects were significantly lower versus children with no effects [16]. In a UK modeling study, an estimated 60% of total cCMV-related costs were indirect costs (i.e., costs incurred personally or by society rather than directly by the public sector), including productivity loss [17]. Similarly, a Japanese modeling study reported that 96% of costs were social costs, including educational costs and patient/parent productivity losses [18]. Family spillover effects are also expected among siblings of children with cCMV, likely due to imbalances in caregiver attention [14].

There is a paucity of data on the family spillover effects of children with cCMV disease in the US and global population. While many studies have examined the short-term costs of cCMV, few have quantified the out-of-pocket economic and health status family spillover effects of caring for a child with cCMV, which are critical to inform the full economic and societal impact of the disease [19].

To fill this evidence gap, a US survey study collected caregiver proxy- and child self-reported data regarding the health status of children with cCMV, as well as family spillover effects, including economic impacts on families and health-status impacts on caregivers and siblings of children with cCMV. The conceptual framework for assessing the lifetime economic burden of cCMV in the US by Lucas et al. 2019 guided our approach [15].

## 2. Materials and Methods

### 2.1. Study Design and Participants

**Figure 1:**
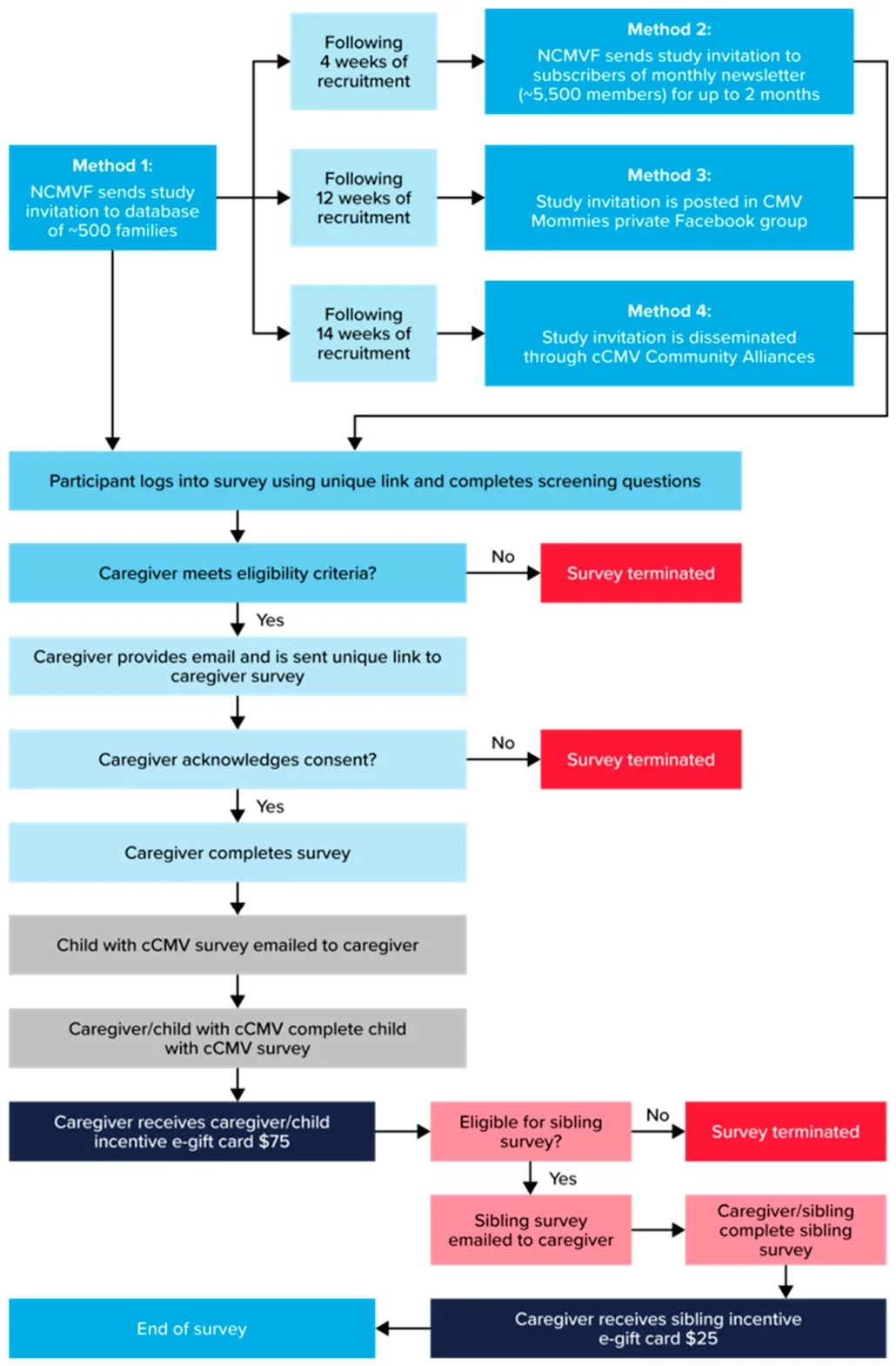
Study design. cCMV = congenital cytomegalovirus; NCMVF = National Cytomegalovirus Foundation.

This non-interventional, cross-sectional, Web-based study included 3 separate surveys to capture the perspectives of children with cCMV and their caregivers and, where appropriate, children with cCMV plus their siblings and caregivers. Survey data were collected from 12 August 2024 through 30 April 2025. The surveys included a background questionnaire on demographic and disease variables, study-specific questions on costs, and multiple standardized questionnaires to assess work productivity and derive health-state utility values. The study design is summarized in Figure 1.

### 2.2. Participant Recruitment and Eligibility

Participants were identified and recruited via the National Cytomegalovirus Foundation (NCMVF) family database, the NCMVF monthly newsletter, a private CMV Facebook group, and cCMV Community Alliances (Figure 1). Caregivers received an email survey invitation followed by the study screener. Those who met the eligibility criteria were sent a unique link to the caregiver survey and, upon completion, a link to the child survey and sibling survey, if applicable. Participants were compensated upon completion of the child survey ($75) and each sibling survey ($25).

#### 2.2.1. Caregivers

Eligible caregivers were required to be aged ≥18 years at study enrollment; a resident of the US; a primary caregiver (i.e., providing care >50% of the time) of only 1 child aged ≤17 years with a caregiver-reported, healthcare professional-based cCMV diagnosis; able to read and respond to an online survey in English; and willing and able to provide informed online consent.

#### 2.2.2. Children with cCMV

Eligible children with cCMV were required to be aged ≤17 years with a caregiver-reported, healthcare professional-based cCMV diagnosis. Parental/legal guardian permission and assent were required for children aged 8–17 years. For children with cCMV aged ≤7 years or who were not capable of completing the survey independently, no additional assent/permission was required, as caregivers completed all survey questions.

#### 2.2.3. Siblings of Children with cCMV

Eligible siblings of children with cCMV were required to be aged ≥4 years, reside in the same household as the child with cCMV, and have no known cognitive or visual disability. Parental/legal guardian permission and assent were required for siblings aged 8–17 years. Siblings aged ≥18 years were required to provide informed consent before completing the survey. For siblings aged ≤7 years, no additional assent/permission was required, as caregivers completed all survey questions.

### 2.3. Demographics

Socioeconomic and demographic questions included gender identity, age, race and ethnicity, type of healthcare insurance for the child with cCMV, employment status (caregivers only), current grade level in school (children with cCMV and siblings only), and household income.

### 2.4. Economic Outcomes

Caregiver-reported economic outcomes included de novo questions (including out-of-pocket and work- or productivity-related costs) and the Work Productivity and Activity Impairment Questionnaire: Specific Health Problem (WPAI:SHP) [20,21,22]. Costs included total annual out-of-pocket expenses (e.g., medications, medical devices, special foods, therapies, transportation and lodging, and personal care), total annual work/productivity-related costs (e.g., hours taken off work and reduced normal working hours), and lifetime out-of-pocket home adaptation and specialized schooling costs (i.e., all one-off costs covering the lifetime of the child with cCMV). The number of hours of work loss reported for an average week was assumed to reflect the average in the past year (i.e., multiplied by 52).

### 2.5. Health-State Utility Outcomes

Caregiver health-state utilities were measured using the EQ-5D-5L [23]. For children with cCMV, no single instrument suitable for use in children aged 0–18 years was available. Therefore, a range of measures was used, including the Infant Health-Related Quality of Life Instrument (IQI) for ages 0–1 years [24], the Health Utilities Preschool (HuPS) instrument for ages 2–4 years [25], and the Health Utilities Index (HUI; both HUI Mark 2 [HUI2] and Mark 3 [HUI3]) for ages 5–17 years [26,27]. An appropriate version of the questionnaire (i.e., caregiver proxy-reported, caregiver-administered, or child-reported) was administered depending on the age and caregiver-reported cognitive ability of the participant. Sibling health-state utilities were collected via the child-friendly EQ-5D-Y-3L (caregiver proxy-reported or self-reported) for those aged 4–17 years or the EQ-5D-5L (self-reported) for those aged ≥18 years [23,28]. Further details of each utility measure are included in Table S1. For all utility outcomes, a score of 1.0 represents full health, 0.0 represents dead, and negative scores are possible for health states that are valued as worse than dead. The US value set was used to calculate all EQ-5D-5L utility index scores (caregivers and siblings) [23]. No US value set was available for the EQ-5D-Y-3L; the German value set was used to calculate all EQ-5D-Y-3L utility index scores (siblings only) [29].

Participants could skip questions they did not feel comfortable answering. Blank surveys are available in Supplementary Materials S1.

### 2.6. Data Management

Data were collected using the Qualtrics Web-based system hosted in a secure private environment. NCMVF did not provide any participant identifier information. Email addresses were collected by RTI Health Solutions (RTI-HS) with permission from eligible caregivers for the purpose of sending links to the survey and providing compensation. Deidentified datasets were provided to the study statisticians for analysis. RTI-HS performed system testing and user acceptance testing of the survey.

### 2.7. Statistical Analysis

The primary objectives of this study are descriptive; no power calculations or formal statistical hypothesis testing was performed. Data were analyzed descriptively using SAS statistical software, version 9.4 (or higher) (SAS Institute Inc., Cary, NC, USA). Missing data were not imputed, and no survey weights were applied to the responses. The extent of missing data for cost calculations was examined, and appropriate methods were applied. Summary statistics for total and disaggregated cost estimates among all families in the study were calculated.

#### Stratification by Severity of Symptoms Associated with cCMV at Birth

Results were stratified by caregiver-reported cCMV symptom severity at birth, which was classified on the basis of the definitions reported by Rawlinson et al. [8]: asymptomatic, asymptomatic with isolated SNHL, mildly symptomatic (only 1 or 2 mild symptoms/sequelae or 1 mild symptom/sequelae plus SNHL), or moderately to severely symptomatic (≥1 moderate to severe symptoms/sequelae, ≥3 mild symptoms/sequelae, or 2 mild symptoms/sequelae plus SNHL) (Table S2). Stratified results should be considered exploratory.

## 3. Results

### 3.1. Participant Demographics and cCMV Clinical Characteristics

The final sample included 227 caregivers, 211 children with cCMV (191 caregiver-completed and 20 child-completed surveys), and 173 siblings (80 caregiver-completed and 93 sibling-completed surveys). Most caregivers (96.9%), children (58.8%), and siblings (50.9%) identified as female (Table 1). Nearly 90% of each group identified as White. The mean age was 36.1 years for caregivers, children with cCMV were mostly aged 2–7 years (45.4%) or 8–17 years (30.8%), and siblings were mostly aged 4–7 years (46.2%) or 8–17 years (49.7%). cCMV symptom severity at birth was mostly moderate to severe (87.7%).

### 3.2. Financial Impact for Caregivers

Most caregivers (81.1%) agreed that caregiving for their child with cCMV had a financial impact on the family. Per family, mean total annual out-of-pocket medical and personal care expenses were $16,577, with occupational therapy, physical therapy, feeding therapy, or speech and language therapy ($9027) largely contributing (Table 2). The mean total work- or productivity-related costs per year were $66,563, which were primarily due to caregiver cessation of work ($33,512). Combined, mean annual out-of-pocket medical and personal care expenses and work- or productivity-related costs totaled $83,140 per family. Mean lifetime out-of-pocket costs (i.e., total costs ever incurred) for home adaptations and specialized schooling were $4713.

### 3.3. Caregiver Work Productivity and Daily Activity Impairment

Among employed caregivers, the mean rate of absenteeism (work time missed) was 13.7% (n = 145), and the mean rate of presenteeism (i.e., impairment while working) was 28.5% (n = 139), with an overall work productivity loss of 33.7% (n = 139) and nonwork daily activity impairment of 34.6% (n = 226). Employed caregivers missed a mean of 5.2 h of work per week due to problems related to their child’s cCMV, versus 1.8 h for other reasons (e.g., holidays or study participation) (Table 2). On a scale of 0 (no impact) to 10 (maximum impact), caregivers gave mean scores of 2.8 and 3.5 for how their child’s condition impacted their work productivity and daily activities, respectively.

### 3.4. Health-State Utility Impact for Caregivers, Children, and Siblings

#### 3.4.1. Caregivers

**Figure 2:**
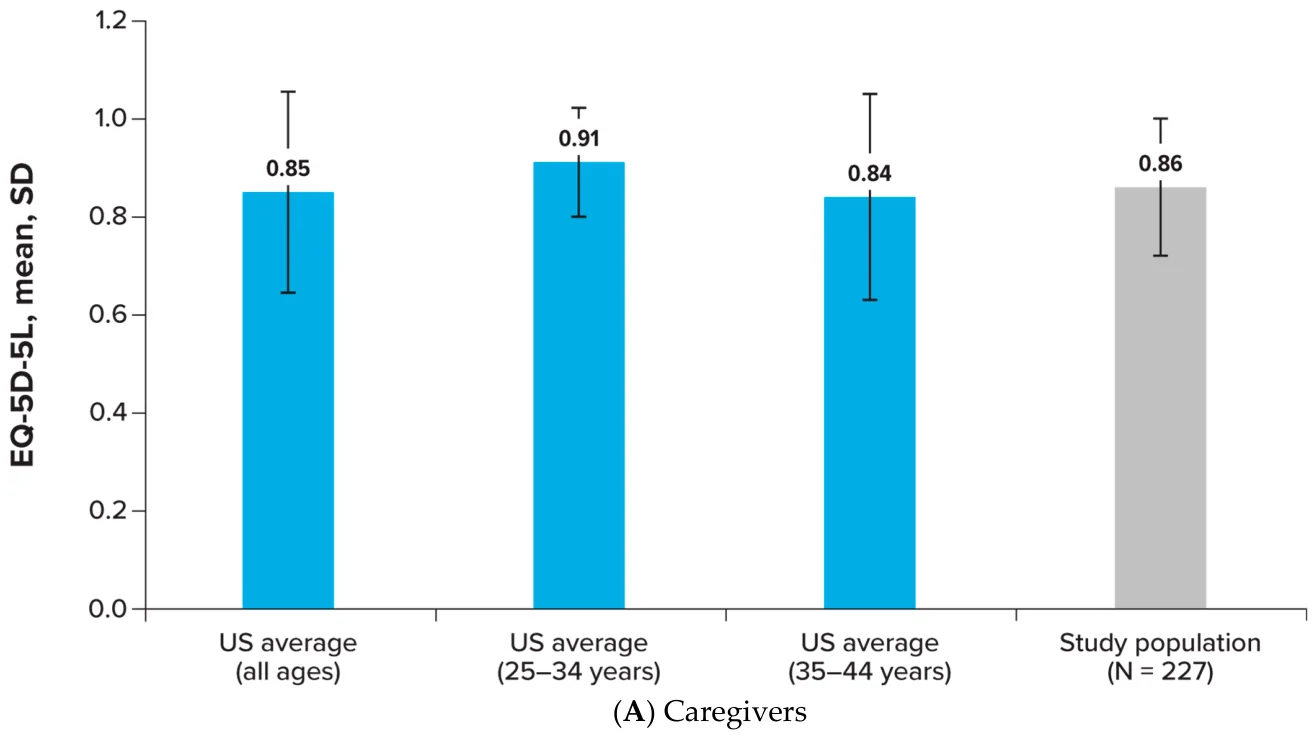

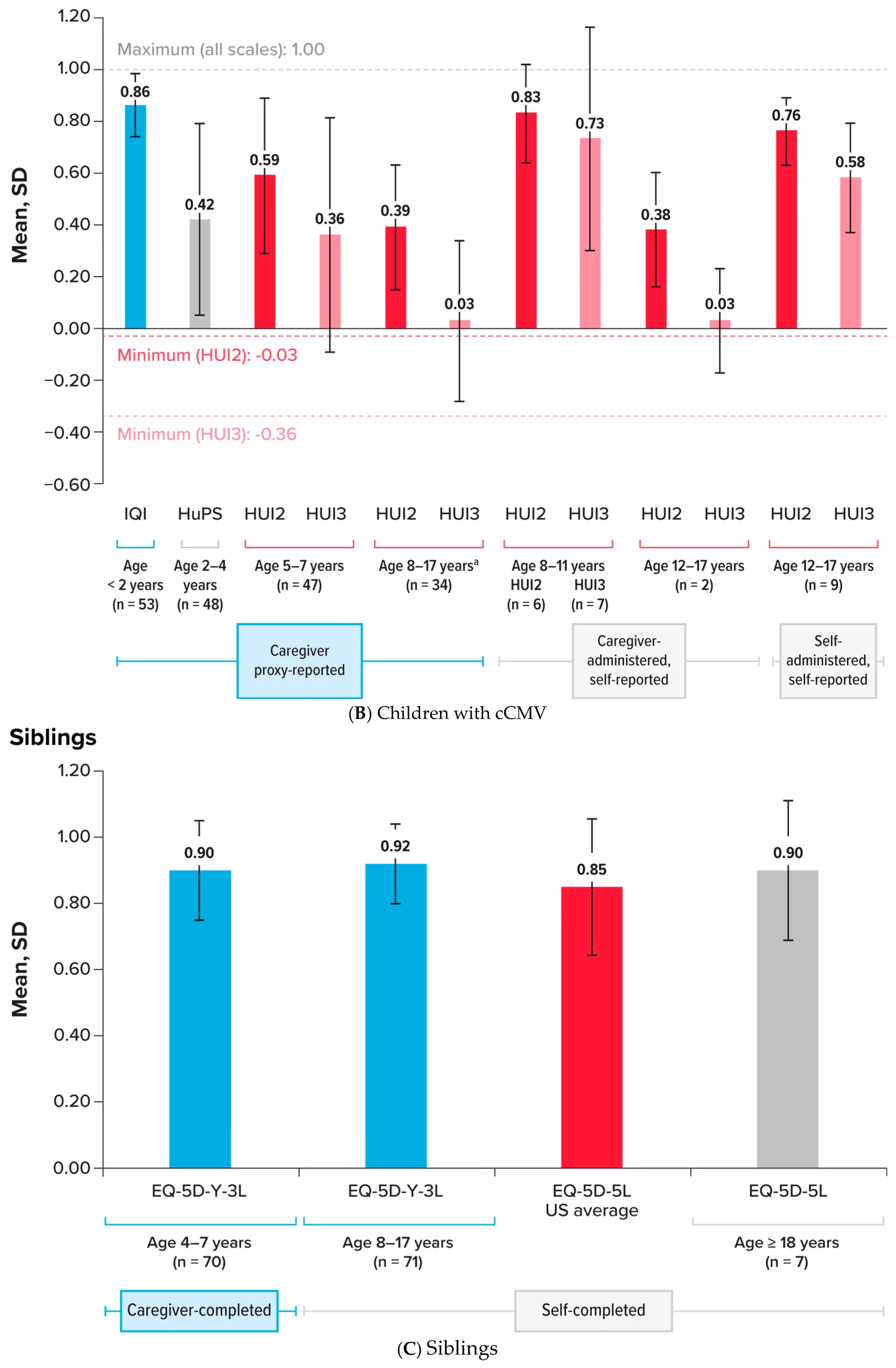
Mean (SD) health-state utilities for (**A**) caregivers, (**B**) children with cCMV, and (**C**) siblings. US averages for EQ-5D-5L were obtained from Jiang et al. 2021 [30]. For HUI2 and HUI3, the lowest possible scores are −0.03 and −0.36, respectively, represented by dotted lines in (**B**) [31]. cCMV = congenital cytomegalovirus; HUI2 = Health Utilities Index Mark 2; HUI3 = Health Utilities Index Mark 3; HuPS = Health Utilities Preschool; IQI = Infant Health-Related Quality of Life Instrument; SD = standard deviation; US = United States.

The mean caregiver EQ-5D-5L utility index score of 0.86 was comparable to the average for the US general population (0.85) and the 35–44-year age group (0.84) but below the 25–34-year age group (0.91), reflecting the mean (standard deviation [SD]) caregiver age of 36.1 (7.0) years (Figure 2) (30). The average caregiver self-rated health at the time of the survey (77.7), on a 0–100 visual analog scale, with 100 being the best health imaginable, was comparable to the US population norm for ages 35–44 years (78.1) but below the norm for ages 25–34 years (84.4) (30).

#### 3.4.2. Children with cCMV

##### Caregiver Proxy-Reported

Health-state utilities among children with cCMV varied across age groups and indices (Figure 2). Among 53 children aged <2 years, the mean (SD) IQI utility score was 0.86 (0.12). Sleep and feeding were frequently affected, with 34.0% of children reported as sleeping well and 43.4% having normal feeding.

Among 48 children aged 2–4 years, the mean (SD) HuPS utility score was 0.42 (0.37). Scores spanned a wide range (−0.21 to 1.00), indicating substantial variation in utility. Speech was one of the most impacted domains: approximately 35% were unable to speak at all, and 20.8% could be fully understood when speaking with people who know them well.

The HUI questionnaire was completed by 47 caregivers of children aged 5–7 years and 34 caregivers of children with developmental delay or disability aged 8–17 years. Among children aged 5–7 years, the mean (SD) HUI2 score was 0.59 (0.30), ranging from 0.11 to 1.00. The mean (SD) HUI3 score was 0.36 (0.45), ranging from −0.31 to 1.00. Speech and memory posed challenges within this age group: 25.0% could not speak at all, and just over half could remember things effectively. For children with cCMV aged 8–17 years, the mean (SD) HUI2 score was 0.39 (0.24), and the HUI3 score was 0.03 (0.31). Ranges for each score extended to near or below 0, underscoring the variability and severity of health-state utility impacts in this population. Notably, approximately one-third of children were nonverbal, over half required equipment or assistance to walk, and 77.1% required help with eating, bathing, dressing, or toileting.

##### Child Self-Reported

Among children aged 8–11 years, the mean (SD) caregiver-administered, self-reported HUI2 score was 0.83 (0.19), and the corresponding HUI3 score was 0.73 (0.43), showing wide variability but clustering toward the higher end of the scale. Two caregivers completed the caregiver-administered, self-reported HUI questionnaire for children aged 12–17 years, with HUI utility scores suggesting limited utility. Among children aged 12–17 years, the mean (SD) self-administered, self-reported HUI2 score was 0.76 (0.13) and the corresponding HUI3 score was 0.58 (0.21).

#### 3.4.3. Siblings

The mean EQ-5D-Y-3L (ages 4–7 years and 8–17 years) and EQ-5D-5L (ages ≥ 18 years) utility scores were 0.90 or higher, indicating generally high perceived health status among siblings of children with cCMV (Figure 2).

### 3.5. Outcomes by Severity of cCMV Sequelae at Birth

Costs varied between cCMV symptom severity groups and were generally lowest for the asymptomatic cCMV at-birth group versus other severity groups (Table S3). Compared with asymptomatic or isolated hearing loss, caregiving for a child classified with mild or moderate to severe cCMV at birth was associated with higher absenteeism, presenteeism, and work productivity loss (Table S3). The caregiver EQ-5D-5L utility index score was lowest for the mild or moderate to severe cCMV at-birth groups among the symptom severity groups (Table S4). Health-state utilities among children with cCMV were also generally lower among groups classified with higher versus lower symptom severity at birth (Table S4). Health-state utilities among siblings of children with cCMV were generally high, with mean scores ranging from 0.83 to 1.00, with no clear trends emerging between cCMV symptom-severity-at-birth groups (Table S4).

## 4. Discussion

This study collected real-world economic outcomes among caregivers of children with cCMV, as well as health-status data describing how cCMV affects children and their families. The results illustrate the substantial and multifaceted economic impacts faced by families of children with cCMV, extending across medical, therapeutic, and daily living expenses. The estimated health-state utility impacts experienced by children with cCMV and their families highlight caregiver presenteeism and activity impairment, varied health utility among children with cCMV, and high perceived health status among siblings.

Mean total annual out-of-pocket medical and personal care expenses and work- or productivity-related costs of $83,140 were reported by caregivers, exceeding a US 2012 estimate for children with disabilities of $30,500 per year per family (including costs directly incurred by families, financial impacts due to impacted family labor supply, the financial impact of disability on children as they age into the labor force, and the costs of safety-net programs) [32]. Annual out-of-pocket medical and personal care expenses averaged $16,577 in the current study, with a notable portion allocated to occupational therapy, physical therapy, feeding therapy, or speech and language therapy ($9027). This is greater than 2016 out-of-pocket medical expense (inpatient costs, outpatient costs, emergency department visits, home health agency costs, and outpatient pharmacy costs) estimates for US families caring for a child with Down syndrome, with annual costs averaging between approximately $2506 for children aged <1 year and $840 for children aged 13–17 years [33]. Conversely, in a 2006 UK study of children with hearing loss, annual costs incurred by families ranged between €230 and €596 in 2001/2002 euros (ranging between $196–$235 and $507–$608, respectively, based on the highest and lowest 2001/2002 US-dollar conversion rates), including the cost of special equipment, special services, and hospital and clinic appointments [34,35,36]. Furthermore, in the present study, lifetime out-of-pocket costs related to one-off payments for home adaptation or specialized schooling presented another layer of economic impact on families of children with cCMV, with a reported average of $4713. Of note, healthcare coverage in the US for a child with cCMV depends mostly on insurance type (e.g., private, public/Medicaid-eligible, or no insurance) but is also impacted by the state of residence, as well as the specific private insurance plan. Allied health is often comprehensively covered in the US, but families frequently pay meaningful out-of-pocket costs for care that exceed visit caps, plus copays and deductibles. Durable medical equipment such as hearing aids and communication devices is generally covered in the US, but house adaptions such as wheelchair ramps and bathroom accessibility renovations are typically excluded.

In this study, the most substantial financial burden on caregivers of children with cCMV was due to work- or productivity-related costs, estimated at $66,563. Moreover, work productivity was notably affected, as demonstrated by the WPAI:SHP results. Caregivers experienced considerable presenteeism and activity impairment, indicating that caregiving responsibilities commonly interfere with caregiver employment and likely contribute to long-term financial strain and altered career trajectories. Beyond these financial impacts on families, costs related to work productivity are also important from a societal perspective by reflecting the overall societal impact of caring for a child with cCMV.

The results of this study also provide a detailed view of the health-state utility impacts experienced by children with cCMV, their caregivers, and their siblings, providing standardized, preference-weighted measures of health states, which can be used in health economic and outcomes research. Caregivers reported generally favorable physical health and functional capacity, as exemplified by the mean EQ-5D-5L utility index score of 0.86, similar to the US general population norm of 0.85 [30]. Health-state utility varied among children with cCMV across ages and survey administration methods, underscoring the heterogeneity of lived health experiences. Notably, some outcomes were comparable to those reported for other serious childhood disabilities. For example, mean caregiver proxy-reported HUI scores among children with cCMV aged 5–7 years (HUI2 = 0.59; HUI3 = 0.36) were similar to those reported for children with Duchenne muscular dystrophy, a progressive and life-limiting condition (mean HUI2 = 0.55; HUI3 = 0.35) [37]. Additionally, published HUI3 scores for UK children aged 5–16 years with conditions such as deafness, Down syndrome, and cerebral palsy (range, 0.12–0.73) fall within the range observed across cCMV symptom severity groups in this study (0.03–0.73) [38]. In contrast, health-state utility scores for siblings were consistently high across age groups, suggesting that observed reductions in utility among children with cCMV reflect disease-specific impacts rather than family-level reporting bias. Importantly, when stratified by cCMV symptom severity at birth, higher costs and lower caregiver and child-with-cCMV health-state utilities were observed with increasing severity, reinforcing the long-term quality-of-life implications of early cCMV manifestations. These findings highlight the substantial and enduring burden of cCMV and emphasize the relevance of health-state utilities for informing policy decisions related to newborn screening, early identification, and targeted intervention.

Furthermore, our findings reinforce prior evidence that a substantial proportion of the overall disease burden associated with cCMV is borne not only by the affected children but also by their families through spillover effects [14]. These spillover impacts arise from prolonged prognostic uncertainty, intensive and sustained caregiving demands, and caregiver psychological distress and manifest in this study as reductions in child health-state utilities, substantial caregiver productivity losses, and considerable out-of-pocket costs. Importantly, such effects are not fully captured by traditional child-centered clinical outcomes or caregiver global health measures alone. Incorporating family spillover effects into economic evaluations is therefore essential for accurately estimating the societal value of cCMV prevention and intervention strategies, including vaccines, maternal antiviral therapies, and newborn screening programs [39]. By quantifying health-state utilities and indirect costs across affected children and family members, this study provides critical input parameters for cost-effectiveness models and national cost-of-illness estimates, thereby strengthening the evidence base needed to inform policy decisions and prioritize investments in cCMV prevention and care.

Future research in this area should utilize multivariable analyses to explore how predictors such as cCMV symptom severity at birth and future sequalae, child demographic characteristics and socioeconomic conditions, and caregiver/household attributes influence family spillover effects associated with cCMV. Additionally, as universal newborn screening (NBS) programs for cCMV expand in the US (e.g., Minnesota implemented statewide universal NBS in February 2023 and Connecticut implemented statewide universal NBS in July 2025), increasing numbers of infants are being diagnosed before symptoms emerge. Among the many opportunities for research that these innovative programs will create, the collection of longitudinal data on the economic burden of cCMV will be critical and allow for examination of how costs evolve over time as children age, as well as whether interventions and modifiable healthcare practices reduce caregiver costs and improve family outcomes.

To the best of our knowledge, this is the first US study to estimate the financial family spillover associated with childhood cCMV, contributing critical evidence toward a more comprehensive understanding of the economic burden of cCMV on families and society. A major strength of this work is its comprehensive characterization of the economic strain experienced by caregivers and families supporting children with cCMV, providing valuable input for future economic evaluations of health technologies for preventing or mitigating the impact of cCMV. Additional strengths include the use of cognitive debriefing interviews to refine survey instruments, the eligibility requirement that caregivers only provided primary care (>50% of the time) to the child with cCMV and did not provide care for another individual with disabilities or medical needs, and procedures to reduce duplicate participation. Recruitment through national advocacy networks enabled enrollment of a heterogeneous sample by geography (families resided in 44 of 50 states) and by child age, gender, race/ethnicity, and disease severity, which is notable, given that cCMV is underdiagnosed, not nationally reportable in the US, and substantially less recognized than other causes of childhood disability [40]. Furthermore, the health status across the pediatric age spectrum was assessed using validated, age-appropriate instruments. The HUI was selected for its sensitivity to domains central to cCMV morbidity, particularly hearing loss, while the HuPS was used for younger children and harmonized with the HUI3 to enable continuous, commensurate utility measurement beginning at age 2 years [25]. This approach allowed for examination of the relationship between classified cCMV symptom severity at birth and subsequent health status and costs, capturing the evolving sequelae that characterize cCMV and remain understudied. Another strength of this research on family spillover effects was the involvement of a mother of a child with cCMV (M.H.P.) in terms of having lived experience with this disease.

These findings should be interpreted alongside several key limitations. The survey was administered in the English language only and relied on a convenience sample of families who elected to participate, with 90% of caregivers identifying as female, White, or having a college-level education, raising the possibility of selection and responder bias, thereby limiting generalizability. Data including cCMV diagnosis and clinical characteristics were caregiver-reported, without medical record verification, introducing potential recall and information bias. The wide age range of participants necessitated the use of multiple utility instruments and modes of administration, which may not be fully comparable despite efforts to harmonize measures. Moreover, because data were collected at a single time point per child and the sample skewed toward younger ages, long-term trajectories and lifetime costs of cCMV were not fully captured, and we were unable to establish causal relationships. The survey also focused on out-of-pocket costs and did not collect any information regarding subsidies by the US government or other agencies. Finally, as a comparator cohort of caregivers with developmentally healthy children was not included in the current study, our results potentially overestimate the true incremental caregiving costs of cCMV [41].

## 5. Conclusions

In this US-based study, childhood cCMV was associated with substantial economic and health-status burden, extending beyond affected children to caregivers and families through family spillover effects. By quantifying health-state utilities and costs across ages and disease severities of children with cCMV, this work provides novel data essential for economic evaluations of cCMV prevention and intervention strategies. These findings underscore the importance of incorporating family-level impacts into policy decisions regarding newborn screening, therapeutic development, and prevention efforts for cCMV.

## Figures and Tables

**Table 1:** Caregiver, child with cCMV, and sibling demographics.

Demographic Variable	Caregiver (N = 227)	Child with cCMV (N = 211) ^a^	Sibling (N = 173)
Age, years			
Mean (SD)	36.1 (7.0)	-	-
Age of child with cCMV, n (%) (N = 227)		-	-
<1 month ^b^	27 (11.9)	-	-
13–24 months ^b^	27 (11.9)	-	-
2–4 years ^b^	51 (22.5)		
5–7 years ^b^	52 (22.9)		
8–17 years^ b^	70 (30.8)	-	-
Age of each sibling (n = 173), ^c^ n (%)		-	-
4–7 years	80 (46.2)	-	-
8–17 years	86 (49.7)	-	-
≥18 years	7 (4.0)	-	-
Gender identity, n (%)			
Female	220 (96.9)	124 (58.8)	88 (50.9)
Male	6 (2.6)	87 (41.2)	81 (46.8)
Prefer not to answer	1 (0.4)	0 (0.0)	4 (2.3)
Race and ethnicity, n (%) ^d,e^			
African American or Black	8 (3.5)	15 (7.1)	8 (4.6)
Alaska Native, American Indian, or Native American	4 (1.8)	4 (1.9)	3 (1.7)
Asian	4 (1.8)	2 (0.9)	0 (0.0)
Hispanic, Latina/Latino, Latine, or Latinx	6 (2.6)	12 (5.7)	6 (3.5)
Native Hawaiian and/or Pacific Islander	1 (0.4)	0 (0.0)	0 (0.0)
White	204 (89.9)	186 (88.2)	151 (87.3)
A race or ethnicity not listed	1 (0.4)	2 (0.9)	0 (0.0)
Prefer not to answer	4 (1.8)	4 (1.9)	13 (7.5)
cCMV symptom severity of child at birth, n (%) ^f^			
Asymptomatic	8 (3.5)	-	-
Isolated hearing loss	7 (3.1)	-	-
Mild	13 (5.7)	-	-
Moderate to severe	199 (87.7)	-	-
Education level, n (%)			
Less than a high school diploma	2 (0.9)	-	-
High school diploma/equivalent or higher or some high school	14 (6.2)	-	-
College degree, associate’s degree, or some college but no degree	128 (56.4)	-	-
Graduate or professional degree or some graduate school but no degree	81 (35.7)	-	-
Prefer not to answer	2 (0.9)	-	-
Health insurance type, n (%) ^d^		-	-
Private	172 (75.8)	-	-
Public (e.g., Medicaid)	81 (35.7)	-	-
Other	10 (4.4)	-	-
No health insurance	3 (1.3)	-	-
Prefer not to answer	2 (0.9)	-	-

cCMV = congenital cytomegalovirus; SD = standard deviation. ^a^ The number of child surveys completed. ^b^ Inclusive. ^c^ The caregiver survey collected sibling age data, but not all caregivers with siblings within the family completed the sibling survey. ^d^ Total percentages > 100% because the survey question stated, “Please select all that apply”. ^e^ The following categories were included in the survey but not selected by participants: “Middle Eastern and/or North African” for race and ethnicity. ^f^ Data on child cCMV symptom severity at birth were collected as part of the caregiver survey.

**Table 2:** Out-of-pocket and work- or productivity-related costs for caregivers and WPAI:SHP item responses for employed caregivers.

Cost Category	Mean (SD) Cost, USD
Annual out-of-pocket medical and personal care expenses	
Medications, hearing aids, medical devices, equipment, or special foods	$2609 ($5773)
Occupational therapy, physical therapy, feeding therapy, or speech and language therapy	$9027 ($21,859)
Other interventional therapies for child with cCMV ^a^	$2911 ($12,400)
Transportation and lodging	$1230 ($2495)
Personal care (including respite care) from a paid provider	$799 ($3955)
Total	$16,577 ($34,089)
Annual work- or productivity-related costs	
Caregiver and other family members’ hours taken off work ^b^	$15,055 ($24,307)
Caregiver reduced normal working hours ^c^	$7659 ($15,212)
Caregiver stopped work ^d^	$33,512 ($32,416)
Family member stopped work ^d^	$10,337 ($23,957)
Total	$66,563 ($61,492)
Lifetime out-of-pocket home adaptation and specialized schooling costs	
Modifications to home environment	$1805 ($7103)
Specialized preschool or daycare	$2510 ($10,932)
Private school (kindergarten through high school)	$399 ($3162)
Total	$4713 ($13,894)
Total annual costs (out-of-pocket medical and personal care expenses and work- or productivity-related costs)	$83,140 ($75,179)
**WPAI:SHP item responses for employed caregivers (N = 154)**	**Mean (SD)**
In the past 7 days	
Hours missed from work because of problems associated with child’s cCMV	5.2 (15.1)
Hours missed from work because of other reasons (e.g., vacation or study participation)	1.8 (6.1)
Hours actually worked	29.6 (18.0)
Effect of child’s cCMV on regular daily activities other than work ^e,f^	3.5 (2.6)
Caregivers who worked in the past 7 days	
Effect of child’s cCMV on work productivity ^e,g^	2.8 (2.3)

cCMV = congenital cytomegalovirus; SD = standard deviation; US = United States; USD = United States dollar; WPAI:SHP = Work Productivity and Activity Impairment Questionnaire: Specific Health Problem. Note: All costs are reported in 2024 USD and were obtained by rounding the analysis results to the nearest dollar. ^a^ For example, horse, aquatic, or music therapies. ^b^ Cost due to work loss within the year was calculated using the reported average weekly number of hours lost multiplied by the US hourly rate according to caregiver age and by 52.14 weeks. ^c^ For caregivers who reduced the number of hours and were currently employed, cost due to reduced working hours was calculated by multiplying the reported hours of work per week before reduction minus their current working hours times 52.14 weeks and the US hourly rate according to caregiver age. ^d^ Cost estimates assume that the caregiver/family member reporting that they had stopped work had not worked during the prior year. ^e^ Using a scale from 0 = no effect to 10 = completely preventing. ^f^ n = 226. ^g^ n = 139.

## Data Availability

Individual-level data reported in this study are not publicly shared. Upon request and subject to review, Moderna (data_sharing@modernatx.com) may provide the deidentified aggregate-level data that support the findings of this study. Deidentified data (including participant data as applicable) may be shared upon approval of an analysis proposal and a signed data access agreement.

## Supplementary Materials

The following supporting information can be downloaded at: https://www.mdpi.com/article/10.3390/v18090973/s1, Supplementary Materials S1. De novo surveys; Table S1. Overview of Health Utility Measures; Table S2. Disease Severity at Birth; Table S3. Out-of-Pocket and Work- or Productivity-Related Costs for Caregivers and Employment Impacts for Caregivers Stratified by cCMV Symptom Severity at Birth; Table S4. Caregiver, Child With cCMV, and Sibling Health-State Utilities Stratified by cCMV Symptom Severity at Birth.
