# Supplementary material for: Economic Burden of Congenital Cytomegalovirus Disease on Families in the United States
Source: Viruses. 2026 Sep 3;18(9):973. doi: 10.3390/v18090973 (PMC13611967; doi:10.3390/v18090973)
Supplement: Supplementary file 1 [file viruses-18-00973-s001.zip › viruses-4473136-supplementary.pdf]

Economic Burden of Congenital Cytomegalovirus Disease on Families in the United States – Supplemental Materials

Supplementary Materials S1. De novo surveys

Family Spillover Impact of Congenital Cytomegalovirus

Primary Caregiver Survey

Informed Consent

[Insert consent form]

Please indicate below if you agree to participate in the current study.

☐ Yes, I agree to participate in this study

☐ No, I do not agree to participate in this study [Terminate survey and display, "You have indicated that you do not agree to participate in the study. Thank you for your time."]

Note: Click [here] for a printable copy of the consent form for your records.

Survey Questions

[Programming note: program on separate page before questions.]

The first set of questions ask about your health-related quality of life.

The second section will be followed by questions about caregiver burden.

The third section of the questionnaire asks you to provide information about your employment status, work productivity, and costs that you have incurred for the care of the child with congenital CMV.

The last section of the survey includes some demographic questions about you.

As a reminder, you can stop the survey at any time and come back to finish it, if needed. You will resume the survey with the last question you answered.

Let's get started!

[PROMIS® Scale v1.2 – Global Health]

[EQ-5D]

[ZARIT BURDEN INTERVIEW]

23. During the past 12 months, have you experienced depression, anxiety, or stress?

☐ Yes

☐ No [Go to Q28]

24. During the past 12 months, were you treated by a professional for depression, anxiety, or stress?

☐ Yes

☐ No [Go to Q26]

25. [If Q24 = Yes] During the past 12 months, how many times were you treated by a professional for depression, anxiety, or stress?

___________ [Include drop-down menu of digits; 1 time, 2 times, 3 times, etc. up to 12, > 12 times] [Go to Q27]

26. [If Q24 = No] Why did you not see a professional to treat your depression, anxiety, or stress? [Go to Q28 after answering]

☐ I did not have time

☐ It was too costly

☐ It was too difficult to get treatment approved by my insurance

☐ I treated it myself

☐ Other reasons

☐ Prefer not to answer

27. [If Q24 = Yes] Are you currently being treated with a prescription drug(s) for depression, anxiety, or stress?

☐ Yes

☐ No

[Work Productivity and Activity Impairment Questionnaire:
Specific Health Problem V2.0 (WPAI:SHP)]

Direct Nonmedical Care Costs

The next set of questions ask about costs associated with caring for a child with congenital CMV.

34. Please rate your level of agreement with the following statement.

"Caregiving for my child with congenital CMV has caused a financial impact on the family."

☐ Strongly agree

☐ Agree

☐ Disagree

☐ Strongly disagree

35. During the past 12 months, on average, how many total hours per day did you and other family members (unpaid caregivers) spend providing care to the child with congenital CMV outside of the time the child was sleeping or attending specialized preschool/education (if applicable)?

___________ hour(s) per day [Allow 2-digit number from 1-24]

36. During an average week, how many total hours have you and other family members had to take off from work to care for your child with congenital CMV? For example, hours missed to take your child to medical appointments, supportive services, or specialized preschool/education; or to provide help with dressing, grooming, eating, or taking medications.

___________ hour(s) in an average week [Allow 3-digit number from 1-168]

37. Have you or someone in your family ever had to stop working to care for your child with congenital CMV?

	Yes	No
You	☐	☐
Someone in your family	☐	☐

38. Have you or someone in your family ever had to turn down a job or promotion to care for your child with congenital CMV?

	Yes	No
You	☐	☐
Someone in your family	☐	☐

39. Have you or someone in your family ever had to change jobs to care for your child with congenital CMV?

	Yes	No
You	☐	☐
Someone in your family	☐	☐

40. [Ask if Q28 = Yes] How many hours per week do you currently work?

____________ hour(s) per week [Allow 3-digit number from 1-168]

41. Have you ever had to reduce your working hours to care for your child with congenital CMV?

☐ Yes [If Q28 = No, go to Q43; if Q28 = Yes, go to Q41a]

☐ No [Go to Q43]

41a. [Ask if Q28 = Yes and Q41 = Yes] Have you ever had to reduce your working hours in your current job to care for your child with congenital CMV?

☐ Yes

☐ No [Go to Q43]

42. [Ask if Q28 = Yes and Q41a = Yes] How many hours per week did you work before reducing your working hours to [insert value from Q40] hours per week?

____________ hour(s) per week [Allow 3-digit number from 1-168; must be > Q40]

Now we will ask you some questions about your child's prescription medications and expenses for congenital CMV.

43. What is the number of different prescription medications your child is currently taking for symptoms related to congenital CMV?

____________ daily medications [Allow 2-digit number from 0-30]

____________ medications taken as needed [Allow 2-digit number from 0-30]

44. During the <u>past 12 months</u>, have you had out-of-pocket costs associated with medications, hearing aids, medical devices, equipment, or special foods needed for your child with congenital CMV (excluding therapies)?

☐ Yes

☐ No [Go to Q46]

45. During the <u>past 12 months</u>, what is the total estimated out-of-pocket cost for medications, hearing aids, medical devices, equipment, or special foods needed for your child with congenital CMV (excluding therapies)?

(Please enter the amount in [US or Canadian] dollars.)

__________ *[US or Canadian]* dollars [Allow 5-digit number from 1 to 99,999]

46. During the <u>past 12 months</u>, have you had out-of-pocket costs associated with occupational therapy, physical therapy, feeding therapy, or speech and language therapy?

☐ Yes

☐ No [Go to Q48]

47. During the <u>past 12 months</u>, what is the total estimated average <u>monthly</u> out-of-pocket cost for these therapies?

(Please enter the amount in [US or Canadian] dollars.)

__________ *[US or Canadian]* dollars [Allow 4-digit number from 1 to 9,999]

48. During the past 12 months, have you had out-of-pocket costs associated with other interventional therapies for your child with congenital CMV (for example, horse, aquatic, or music therapies)?

- ☐ Yes
- ☐ No [Go to Q50]

49. During the past 12 months, what is the total estimated average monthly out-of-pocket cost for these therapies?

(Please enter the amount in [US or Canadian] dollars.)

__________ *[US or Canadian]* dollars [Allow 4-digit number from 1 to 9,999]

50. During the past 12 months, approximately how much have you or your family spent on transportation and lodging associated with your child's congenital CMV? This includes transportation to and from a physician office, hospital, or specialty care center (including gas and parking fees) and the cost of lodging for a visit outside the local area.

(Please enter the amount in [US or Canadian] dollars.)

__________ *[US or Canadian]* dollars [Allow 5-digit number from 0 to 99,999]

51. Have you ever modified your home environment (for example, installed special alarms, ramps, or railings) or moved your residence to accommodate the congenital CMV-related disability of your child?

- ☐ Yes, I made modifications to my current home
- ☐ Yes, I moved to a home that can accommodate the congenital CMV-related disability of my child [Go to Q53]
- ☐ No [Go to Q53] [Exclusive]

52. [Display if Q51 ("Yes, I made modifications…") is selected] What was the estimated total cost of the modifications you made to your home environment to accommodate the congenital CMV-related disability of your child?

(Please enter the amount in [US or Canadian] dollars.)

__________ *[US or Canadian]* dollars [Allow 6-digit number from 1 to 999,999]

53. Has your child ever attended a specialized preschool or daycare that was designed to accommodate the congenital CMV-related disability of your child?

- ☐ Yes
- ☐ No [Go to Q56]

54. For how many years has your child attended a specialized preschool or daycare?

__________ year(s) [Allow 2-digit number from 1-12]

55. On average, what was your estimated out-of-pocket cost for tuition per year for the specialized preschool or daycare?

(Please enter the amount in [US or Canadian] dollars.)

__________ *[US or Canadian]* dollars [Allow 5-digit number from 1 to 99,999]

56. [Skip to Q59 if S8 < 5] [Display if S8 (age of child with cCMV) ≥ 5] Has your child ever attended a private school for kindergarten through high school because of congenital CMV and for which you had to pay tuition out of pocket?

- ☐ Yes
- ☐ No [Go to Q59]

57. [Display if S8 (age of child with cCMV) ≥ 5 and Q56 = Yes] For how many years has your child attended a private school?

__________ year(s) [Allow 2-digit number from 1-12]

58. [Display if S8 (age of child with cCMV) ≥ 5 and Q56 = Yes] On average, what was your estimated out-of-pocket cost for tuition per year for the private school?

(Please enter the amount in [US or Canadian] dollars.)

__________ *[US or Canadian]* dollars [Allow 5-digit number from 1 to 99,999]

59. Has your child ever received classroom accommodations to meet their educational needs for congenital CMV-related disabilities?

☐ Yes

☐ No

60. Does your child receive personal care (including respite care) from a paid provider?

☐ Yes

☐ No [Go to Q63]

61. During the past 12 months, approximately how many hours per week has your child received personal care from a paid provider?

__________ hour(s) [Allow 3-digit number from 1-168]

62. During the past 12 months, approximately how much have you or your family spent out of pocket on personal care for your child?

(Please enter the amount in [US or Canadian] dollars.)

__________ *[US or Canadian]* dollars [Allow 5-digit number from 1 to 99,999]

Open-Ended Question

63. Is there anything additional that you would like to share about your experience caring for a child with congenital CMV?

☐ Yes [Provide space for open-ended response; allow up to 500 characters]

☐ No

Demographics

The last part of the survey asks questions about you.

64. What gender identity best describes you?

- ☐ Female
- ☐ Male
- ☐ Gender fluid/nonbinary/a gender identity not listed
- ☐ Prefer not to answer

65. What race(s) or ethnicity(ies) do you consider yourself to be? (Please select all that apply.)

[If S2 = United States]

- ☐ African American or Black
- ☐ Alaska Native, American Indian, or Native American
- ☐ Asian or Asian American
- ☐ Hispanic, Latina/Latino, Latine, or Latinx
- ☐ Middle Eastern and/or North African
- ☐ Native Hawaiian and/or Pacific Islander
- ☐ White
- ☐ A race or ethnicity not listed
- ☐ Prefer not to answer [Exclusive]

[If S2 = Canada]

- ☐ Arab
- ☐ Black
- ☐ Chinese
- ☐ Filipino
- ☐ Indigenous (for example, member of a First Nation, Inuit, or Métis organization)
- ☐ Japanese
- ☐ Latin American
- ☐ South Asian (for example, East Indian, Pakistani, Sri Lankan)
- ☐ Southeast Asian (for example, Vietnamese, Cambodian, Laotian, Thai)
- ☐ West Asian (for example, Iranian, Afghan)
- ☐ White
- ☐ A race or ethnicity not listed
- ☐ Prefer not to answer [Exclusive]

66. What is the highest level of education you have completed?

- ☐ Less than high school
- ☐ Some high school
- ☐ High school diploma or equivalent (for example, GED)
- ☐ Some college but no degree
- ☐ Associate's degree (2-year college degree)
- ☐ College degree (for example, BA, BS)
- ☐ Some graduate school but no degree
- ☐ Graduate or professional degree (for example, MBA, MS, MD, PhD)
- ☐ Prefer not to answer

67. What is your current health insurance coverage?

Please select all that apply.

[If S2 = United States]

- ☐ Insurance through a current or former employer or union (or another family member's employer or union)
- ☐ Insurance purchased directly from an insurance company
- ☐ Medicare or Medicare Advantage Plan, for people 65 and older, or people with certain disabilities
- ☐ Medicaid, Medical Assistance, or any kind of government-assistance plan for those with low incomes or a disability
- ☐ TRICARE or other military healthcare
- ☐ VA
- ☐ Indian Health Service
- ☐ Other
- ☐ No health insurance [Exclusive]
- ☐ I am not sure [Exclusive]
- ☐ Prefer not to answer [Exclusive]

[If S2 = Canada]

- ☐ Publicly funded health care
- ☐ Private insurance through a current or former employer (or another family member's employer)
- ☐ Insurance purchased directly from an insurance company
- ☐ Other
- ☐ No health insurance [Exclusive]
- ☐ I am not sure [Exclusive]
- ☐ Prefer not to answer [Exclusive]

68. [If S2 = United States] Please select the state in which you currently live.

__________ [Drop-down menu that includes all 50 states and Washington, DC.]

[If S2 = Canada] Please select the province or territory in which you currently live.

__________ [Drop-down menu that includes all Canadian provinces/territories: Alberta, British Columbia, Manitoba, New Brunswick, Newfoundland and Labrador, Northwest Territories, Nova Scotia, Nunavut, Ontario, Prince Edward Island, Quebec, Saskatchewan, Yukon]

69. How would you describe the area where you currently live?

- ☐ Urban (a city or metropolitan area)
- ☐ Suburban (a large residential area that surrounds main cities)
- ☐ Rural

70. Which of the following best describes your employment status?

- ☐ Employed full time
- ☐ Employed part time
- ☐ Self-employed
- ☐ Not employed but looking for work
- ☐ Not employed and not looking for work
- ☐ Retired
- ☐ Prefer not to answer

71. Approximately how much was your total household income before taxes last year, including overtime pay, tips, interest, commissions, etc.?

- ☐ Less than $25,000
- ☐ $25,000 to $49,999
- ☐ $50,000 to $99,999
- ☐ $100,000 to $149,999
- ☐ $150,000 or more
- ☐ Prefer not to answer

72. What is your current marital status?

- ☐ Single, never married
- ☐ Married or in a domestic partnership
- ☐ Widowed
- ☐ Divorced with primary custody
- ☐ Divorced with shared custody
- ☐ Separated with primary custody
- ☐ Separated with shared custody
- ☐ Prefer not to answer

73. What is the total number of adults, including yourself, older than 18 years living in your household? [Include drop-down menu of digits 1-6, > 6]

74. What is the total number of children, other than the child with congenital CMV, younger than 18 years, living in your household? [Include drop-down menu of digits 0-6, > 6]

75. Does the child with congenital CMV have 1 or more siblings who live in the same household?

- ☐ Yes
- ☐ No [If S8 ≥ 8 years, go to Q78; if S8 ≤ 7 years, survey is complete. Go to Q87 if recruited via NCMVF. Otherwise, end survey and display "Thank you for completing this survey. You will receive an email with a link to the survey about the child with congenital CMV."]

76. [If Q75 = Yes] How many siblings? [Allow 1-digit number from 1-6]

77. What is the age of each sibling? [Q77 is asked before Q81 (in the sibling section) below for the number of siblings indicated in Q76] [Required]

- ☐ 0-12 months
- ☐ 13-24 months

__________ years [Allow 2-digit number 2-99]

Confirmation of Age and Abilities of Child With cCMV [S8 ≥ 8 years]

Thank you for completing the questions about your experience caring for a child with congenital CMV.

The next portion of the study is a separate survey that is about your child's health-related quality of life and how congenital CMV affects it.

Some of the questions will be answered by your child or by you, if your child is too young or is unable to complete the questionnaire independently. Please answer the following questions to determine which version of the questionnaire is appropriate for your child with congenital CMV.

78. Does your child with congenital CMV have a developmental delay or disability that may make it hard for them to complete the questionnaire independently? [Required]

- ☐ Yes, cognitive delays/intellectual disability [Caregiver will complete the proxy HRQOL and utility measures]
- ☐ Yes, vision loss, motor limitations, or another disability that makes it difficult to complete the questionnaire independently [Caregiver will read the HRQOL and

utility measure questions to the child and record the child's responses in the survey]

☐ No, my child does not have a disability that would make it difficult for them to complete the questionnaire independently [Child will complete the HRQOL and utility measure]

79. [If S8 = No and Q78 = No] You indicated previously that you are not the legally authorized representative of your child with congenital CMV. For your child to participate and complete the questionnaire, we must have permission from the child's legally authorized representative.
Please provide the email address for the child's legally authorized representative. We will send an email with the permission form for the legally authorized representative to complete. Once their permission is received, we will send a link to the questionnaire for the child to complete.

Please feel confident that this information will not be used to contact the legally authorized representative for any reason other than to send the permission form.

Please enter the email address for the child's legally authorized representative:	
Please confirm the email address for the child's legally authorized representative:	

80. [If S8 = No and Q78 = No] Should the questionnaire that the child with congenital CMV will complete be emailed to the legally authorized representative at [insert email from Q79] or to you?

☐ Email the survey link to the child's legally authorized representative

☐ Email the survey link to me

Confirmation of Age and Abilities of Sibling [If Q75 = Yes and Q77 ≥ 4]

As part of this study, we are interested in learning more about siblings' experiences living with a brother or sister who has congenital CMV. Some of the questions will be answered by the sibling or by you if the sibling is too young or would not be able to answer the questionnaire independently. If you are interested in participating, we will send you an email with a link to the sibling survey. It can be completed whenever it is convenient to you and the sibling.

81. [Ask for each sibling in Q77 is 4 to 7 years] Would you be interested in completing a brief questionnaire about your child's (aged [insert from Q77] years) experiences living with a brother or sister with congenital CMV? [Required]

- ☐ Yes
- ☐ No

82. [Ask for each sibling in Q77 ≥ 8] Would the sibling (aged [insert from Q77] years) be interested in completing a brief questionnaire about living with a brother or sister with congenital CMV? [Required]

- ☐ Yes
- ☐ No

83. [If Q77 is 8 to 17 years and Q82 = Yes] Are you the legally authorized representative of the sibling (aged [insert from Q77] years)? [Required]

- ☐ Yes
- ☐ No [Go to End of Survey]

84. [If Q77 is 8 to 17 years and Q82 = Yes] Does the sibling (aged [insert from Q77] years) have a developmental delay or disability that may make it hard for them to complete the questionnaire independently? [Required]

- ☐ Yes, cognitive delays/intellectual disability [Sibling not eligible]
- ☐ Yes, vision loss, motor limitations, or another disability that makes it difficult to complete the questionnaire independently [Sibling not eligible]

☐ No, the sibling does not have a disability that would make it difficult for them to complete the questionnaire independently [Eligible; Sibling will complete the survey]

85. [If Q82 = Yes and Q83 = No and Q84 = No] You indicated that you are not the legally authorized representative of the sibling (aged [insert from Q77] years). In order for the sibling (aged [insert from Q77] years) to participate and complete the questionnaire, we must have permission from the sibling's legally authorized representative.
Please provide the email address for the sibling's legally authorized representative. We will send an email with the permission form for the legally authorized representative to complete. Once their permission is received, we will send a link to the questionnaire for the sibling to complete.

Please understand that this information will not be used to contact the legally authorized representative for any reason other than to send the permission form. [Required]

Please enter the email address for the sibling's (aged [insert from Q77] years) legally authorized representative:	
Please confirm the email address for the sibling's (aged [insert from Q77] years) legally authorized representative:	

86. [If Q82 = Yes and Q83 = No and Q84 = No] Should the questionnaire that the sibling (aged [insert from Q77] years) will complete be emailed to the legally authorized representative at [insert email from Q85] or to you? [Required]

☐ Email the survey link to the child's legally authorized representative

☐ Email the survey link to me

[If recruited by NCMVF, go to next section. If recruited by CMV Canada, end survey. Display: "Thank you for completing the caregiver survey. An email with a link to the survey about the child with congenital CMV will be sent separately. A separate email will be sent for each sibling who is eligible to complete the sibling survey. Please allow a few minutes to receive each email. You may close this screen."]

Interest in Future NCMVF Studies [For all participants recruited by NCMVF]

87. Are you interested in participating in future research studies conducted by the National CMV Foundation (NCMVF)?

☐ Yes

☐ No [Go to End of Survey]

88. [If Q87 = Yes] Do you give your permission for RTI Health Solutions to provide your email address to NCMVF? The NCMVF will only use your email address to contact you about future research studies about congenital CMV.

☐ Yes

☐ No

89. [If Q88 = Yes] Please confirm the email address that can be provided to NCMVF.

Please enter your email address: ______________________

Please confirm your email address: ______________________

[End survey. Display "Thank you for completing the caregiver survey. An email with a link to the survey about the child with congenital CMV will be sent separately. A separate email will be sent for each sibling who is eligible to complete the sibling survey. Please allow a few minutes to receive each email. You may close this screen."]

Family Spillover Impact of Congenital Cytomegalovirus

cCMV Child Survey

[If CG S8 < 8 years, go to Survey questions and ask caregiver-completed prompts]

[If CG S8 ≥ 8 years and CG Q78 = either Yes option, go to Survey Questions and ask caregiver-completed prompts]

[If CG S8 ≥ 8 years, CG S7 = Yes, and CG Q78 = No, go to Parental Agreement and ask child-completed prompts]

[If CG S8 ≥ 8 years, CG S7 = No, and CG Q78 = No; a separate parental agreement will be emailed to the email provided in CG Q79. Once parental permission is received, end survey and display the following message: "Thank you for providing permission. An email with a link to the survey for the child with congenital CMV will be sent separately. Please allow a few minutes to receive the email. You may close this screen." A separate email will be sent to the child survey based on response to CG Q80. Once accessed, the survey will begin with Introduction section. Ask child-completed prompts.]

**Parental Agreement** [Only if CG S8 ≥ 8 years and CG S7 = Yes; will be emailed separately if CG S7 = No]

[INSERT PARENTAL PERMISSION FORM]

Do you give your permission for your child to participate in this study?

- ☐ Yes
- ☐ No [Terminate and display, "Unfortunately, based on your response, your child is not eligible to complete the survey. Thank you for your time and interest."]

Note: Click [here] for a printable copy of the permission form for your records.

**Introduction** [only if CG S8 ≥ 8 years and CG Q78 = No]

The following questionnaire was designed for your child to answer the questions. We will first ask your child whether they want to participate in the study. Once they provide their agreement to participate, we will proceed to the questions.

Please do not provide the answers for your child completing this questionnaire. It is important that we get this information directly from your child so that we can understand what matters to them. However, you may assist your child as needed with a specific word or phrase.

At this time, please allow your child to complete the remainder of the questionnaire.

Minor Agreement [only if CG S8 ≥ 8 years and CG Q78 = No]

[INSERT ASSENT FORM]

Would you like to participate in this study?

- ☐ Yes
- ☐ No [Terminate and display, "Unfortunately, based on your responses, you are not eligible to complete the survey. Thank you again for your time and interest."]

Survey Questions

[CG S8 < 8 years or CG S8 ≥ 8 years and CG Q78 = Yes] The first set of questions ask about the things that are important to your child with congenital CMV for their health, so they can go to school and do activities with family and friends. This section will be followed by questions about your child's experiences living with congenital CMV. The last section includes some questions to describe your child with congenital CMV. Let's get started!

[CG S8 ≥ 8 years and CG Q78 = No] The first set of questions ask about the things that are important to you for your health so you can go to school and do activities with your family and friends. This section will be followed with questions about living with congenital CMV. The last section includes some questions about you. Let's get started!

Health-Related Quality of Life

[Insert PRO instrument based on CG S2, CG S8, and CG Q78]

Instrument	Version
PedsQL	Aged < 12 months: PedsQL v1.0 Generic Core Scales: Parent Report for Infants (ages 1-12 months) (completed by caregiver)
	Aged 13 to < 24 months: PedsQL v3.0 Generic Core Scales: Parent Report for Infants (ages 13-24 months) (completed by caregiver)
	Aged 2 to 4 years: PedsQL v4.0 Generic Core Scales: Parent Report for Toddlers (ages 2-4) (completed by caregiver)
	Aged 5 to 7 years: PedsQL v4.0 Generic Core Scales: Parent Report for Young Children (ages 5-7) (completed by caregiver)
	Aged 8 to 12 years, no disabilities: PedsQL v4.0 Generic Core Scales: Child Report (ages 8-12) (self-completed by child)
	Aged 8 to 12 years, with cognitive disability: PedsQL v4.0 Generic Core Scales: Parent Report for Children (ages 8-12) (completed by caregiver)
	Aged 8 to 12 years, with visual disability, no cognitive disability: PedsQL v4.0 Generic Core Scales: Child Report (ages 8-12) (read to child, recorded by caregiver)
	Aged 13 to 17 years, no disabilities: PedsQL v4.0 Generic Core Scales: Teen Report (ages 13-18) (self-completed by child)
	Aged 13 to 17 years, with cognitive disability: PedsQL v4.0 Generic Core Scales: Parent Report for Teens (ages 13-18) (completed by caregiver)
	Aged 13 to 17 years, with visual disability, no cognitive disability: PedsQL v4.0 Generic Core Scales: Teen Report (ages 13-18) (read to child, recorded by caregiver)
Neuro-QOL	Aged 5 to 7 years (completed by caregiver)
	Aged 8 to 17 years, no disabilities (self-completed by child)
	Aged 8 to 17 years, with cognitive disability (completed by caregiver)
	Aged 8 to 17 years, with visual disability, no cognitive disability (read to child, recorded by caregiver)
HEAR-QL [If CG S12 "Hearing loss" = Yes]	Aged 7 years: HEAR-QL in Children (7-12 years old) (completed by caregiver)
	Aged 8 to 12 years, no disabilities: HEAR-QL in Children (7-12 years old) (self-completed by child)

Instrument	Version
	Aged 8 to 12 years, with cognitive or visual disabilities: HEAR-QL in Children (7-12 years old) (completed by caregiver)
	Aged 13 to 17 years, no disabilities: HEAR-QL in Adolescents (13-18 years old) (self-completed by child)
	Aged 13 to 17 years with cognitive or visual disabilities: HEAR-QL in Adolescents (13-18 years old) (completed by caregiver)

[For Neuro-QoL: Instructions should read: Please select [mark] one response for each question or statement.]

[For HEAR-QL: Instructions should read: Please click on the response choice that best answers each question for you…]

Utility

[Insert appropriate utility instrument based on CG S8 and CG Q78]

Instrument	Version
IQI	Aged 0 to < 24 months (completed by caregiver)
HuPS	Aged 2 to 4 years (completed by caregiver)
HUI	Aged 5 to 7 years (completed by caregiver)
	Aged 8 to 17 years, with cognitive disability (completed by caregiver)
	Aged 8 to 11 years, no cognitive disability (read to child, recorded by caregiver)
	Aged 12 to 17 years, with visual disability, no cognitive disability (read to child, recorded by caregiver)
	Aged 12 to 17 years, no cognitive or visual disabilities (self-completed by child)

[If CG S8 = 8-11 and CG Q78 = No:

Before the HUI2 display: The next set of questions are designed for an adult to read them to you. The adult will read you the question and record your answer. Please ask your parent or caregiver to read the questions to you. Once you finish this set of questions, you can finish the survey on your own.

After the HUI2, display: This finishes the questions that need to be read by a parent or caregiver. The child can read and answer the rest of the questions on their own.]

Open-Ended Question

1. *[If caregiver-completed]* Is there anything additional that you would like to share about your child's experience living with congenital CMV?
   [If child/self-completed] Is there anything that you would like to share about living with congenital CMV?

   ☐ Yes [Provide space for open-ended response]

   ☐ No

Demographics

[If caregiver-completed] This next part of the questionnaire will ask questions about your child with congenital CMV.

[If child/self-completed] This next part of the questionnaire will ask questions about you.

2. *[If caregiver-completed]* What gender identity best describes your child?
   [If child/self-completed] What gender identity best describes you?

   ☐ Female

   ☐ Male

   ☐ Gender fluid/nonbinary/a gender identity not listed

   ☐ Prefer not to answer

3. *[If caregiver-completed]* What race(s) or ethnicity(ies) do you consider your child to be? Please select all that apply.
   [If child/self-completed] What race(s) or ethnicity(ies) do you consider yourself to be? Please select all that apply.

   [If CG S2 = United States]

   ☐ African American or Black

   ☐ Alaska Native, American Indian, or Native American

   ☐ Asian or Asian American

   ☐ Hispanic, Latina/Latino, Latine, or Latinx

   ☐ Middle Eastern and/or North African

   ☐ Native Hawaiian and/or Pacific Islander

   ☐ White

   ☐ A race or ethnicity not listed

   ☐ Prefer not to answer [Exclusive]

[If CG S2 = Canada]

- ☐ Arab
- ☐ Black
- ☐ Chinese
- ☐ Filipino
- ☐ Korean
- ☐ Japanese
- ☐ Latin American
- ☐ Member of a First Nation, Inuit, or Métis organization
- ☐ South Asian (for example, East Indian, Pakistani, Sri Lankan)
- ☐ Southeast Asian (for example, Vietnamese, Cambodian, Laotian, Thai)
- ☐ West Asian (for example, Iranian, Afghan)
- ☐ White
- ☐ A race or ethnicity not listed
- ☐ Prefer not to answer [Exclusive]

4. *[If caregiver-completed]* What is your child's current grade in school?

- ☐ Not enrolled in school
- ☐ Preschool
- ☐ Pre-kindergarten
- ☐ Kindergarten
- ☐ 1
- ☐ 2
- ☐ 3
- ☐ 4
- ☐ 5
- ☐ 6
- ☐ 7
- ☐ 8
- ☐ 9

- ☐ 10
- ☐ 11
- ☐ 12
- ☐ Graduated high school

[If child/self-completed] What is your current grade in school?

- ☐ I do not go to school
- ☐ 1
- ☐ 2
- ☐ 3
- ☐ 4
- ☐ 5
- ☐ 6
- ☐ 7
- ☐ 8
- ☐ 9
- ☐ 10
- ☐ 11
- ☐ 12
- ☐ Graduated high school

5. [If child/self-completed] **Please select the best response.**

- ☐ I completed the questionnaire by myself
- ☐ I completed the questionnaire with help from an adult
- ☐ Someone else completed the questionnaire for me

Thank You Screen

Thank you for taking the time to complete this survey.
Please take note of the following number that can be used for future reference:

Please click Next to receive information about the incentive.

Incentive Landing Screen

As a token of our appreciation, your family will receive a $[CG S2 = US, insert 75; CG S2 = Canada, insert 100] e-gift card for completing this survey.

The email will be sent from Rybbon, an online e-gift card company, and will be sent to the email that was previously provided.

The subject of the email will be “Gift Card for Completion of Surveys”; please check your spam folder.

You may close this screen.

Family Spillover Impact of Congenital Cytomegalovirus

Sibling Survey

[If CG Q77 < 8 years and CG Q81 = Yes, go to Survey questions and ask caregiver-completed prompts]

[If CG Q77 = 8 to 17 years, CG Q82 = Yes, CG Q83 = Yes, and CG Q84 = No; go to Parental Agreement and ask sibling-completed prompts]

[If CG Q77 ≥ 18 years and CG Q82 = Yes, go to Informed Consent and ask sibling-completed prompts]

[If CG Q77 = 8 to 17 years, CG Q82 = Yes, CG Q83 = Yes, and CG Q84 = No; a separate parental agreement will be emailed to the email provided in CG Q85. Once parental permission is received, end survey and display the following message: "Thank you for providing permission. An email with a link to the survey for the sibling of the child with congenital CMV will be sent separately. Please allow a few minutes to receive the email. You may close this screen." A separate email will be sent to the sibling survey based on response to CG Q86. Once accessed, the survey will begin with Introduction section. Ask sibling-completed prompts.]

Parental Agreement [Only if CG Q77 = 8 to 17 years and CG Q82 = Yes, CG Q83 = Yes, and CG Q84 = No
If CG Q82 = Yes, CG Q83 = No, and CG Q84 = No, will be sent separately to email address in CG Q85]

[INSERT PARENTAL PERMISSION FORM]

Do you give your permission for your child to participate in this study?

☐ Yes

☐ No [Terminate and display, "Unfortunately, based on your response, your child is not eligible to complete the survey. Thank you for your time and interest."]

Note: Click [here] for a printable copy of the permission form for your records.

Introduction [only if CG Q77 = 8 to 17 years]

The following questionnaire was designed for the sibling who is 8 to 17 years old to answer the questions. We will first ask the sibling whether they want to participate in the study. Once they provide their agreement to participate, we will proceed to the questions.

Please do not provide the answers for the sibling completing this questionnaire. It is important that we get this information directly from the sibling so that we can understand what matters to them. However, you may assist the sibling as needed with a specific word or phrase.

At this time, please allow the sibling to complete the remainder of the questionnaire.

Minor Agreement [only if CG Q77 = 8 to 17 years, CG Q82 = Yes, and CG Q84 = No]

[INSERT MINOR SIBLING ASSENT FORM]

Would you like to participate in the study?

☐ Yes

☐ No [Terminate and display, "Unfortunately, based on your responses, you are not eligible to complete the survey. Thank you again for your time and interest."]

Note: Click [here] for a printable copy of the assent form for your records.

Informed Consent [only if CG Q77 ≥ 18 years and CG Q82 = Yes]

The following questionnaire was designed for the sibling who is aged 18 years or older to answer the questions. At this time, please allow the sibling to complete the remainder of the questionnaire. We will begin by obtaining their agreement to participate in the study. They will then answer the questions.

[INSERT ADULT SIBLING CONSENT FORM]

Would you like to take part in this study?

☐ Yes, I agree to participate in the study

☐ No, I do not agree to participate in the study [Terminate and display, "Unfortunately, based on your responses, you are not eligible to complete the survey. Thank you again for your time and interest."]

Note: Click [here] for a printable copy of the consent form for your records.

Survey Questions

[CG Q77 = 4 to 7 years: caregiver-completed] Please answer all the questions to the best of your ability, even if you are unsure about an answer or feel that an item does not apply to the sibling.

The first set of questions ask about the things that are important to the sibling's health so they can go to school and do activities with family and friends. This section will be followed by questions about living with a brother or sister who has congenital CMV. The last section includes some questions about the sibling.

When completing the questionnaire, please think about how the health of the child with cCMV impacts their brother's or sister's life.

Let's get started!

[CG Q77 = 8 to 17 years and CG Q84 = No: self-completed by sibling] Please answer all the questions in the questionnaire to the best of your ability, even if you are unsure about an answer or feel that an item does not apply to you.

If you are unsure of a word or phrase, you can ask an adult for help on a question if you need it.

The first set of questions ask about the things that are important to you for your health so you can go to school and do activities with your family and friends. This section will be followed by questions about living with a brother or sister who has congenital CMV. The last section includes some questions about you.

When completing the questionnaire, please think about and include any impact that your brother's or sister's health has on your life.

Let's get started!

[CG Q77 = ≥ 18 years: self-completed by sibling] The first set of questions ask about the things that are important to you for your health so you can go to school or work and do activities with your family and friends. This section will be followed by questions about living with a brother or sister who has congenital CMV. The last section includes some questions about you.

When completing the questionnaire, please think about and include any impact that your brother's or sister's health has on your life.

Let's get started!

Health-Related Quality of Life

[Insert PRO instrument based on CG S2 and CG Q77]

Instrument	Version
PedsQL	Aged 4 years: PedsQL v4.0 Generic Core Scales: Parent Report for Toddlers (ages 2-4) (completed by caregiver)
	Aged 5 to 7 years: PedsQL v4.0 Generic Core Scales: Parent Report for Young Children (ages 5-7) (completed by caregiver)
	Aged 8 to 12 years: PedsQL v4.0 Generic Core Scales: Child Report (ages 8-12) (self-completed by sibling)
	Aged 13 to 17 years: PedsQL v4.0 Generic Core Scales: Teen Report (ages 13-18) (self-completed by sibling)
PROMIS Global Health	Aged 18 years and older: PROMIS v1.2 – Global Health (self-completed by sibling)

[If CG Q77 ≥ 18 years: For PROMIS GH, use the following instructions: The following questions ask about your overall health. Please select [mark] one response for each question or statement.]

Utility

[Insert utility instrument based on CG S2 and CG Q77]

Instrument	Version
EQ-5D-Y-3L proxy version 2	Aged 4 to 7 years (completed by caregiver)
EQ-5D-Y-3L	Aged 8 to 17 years (self-completed by sibling)
EQ-5D-5L	Aged 18 years and older (self-completed by sibling)

Open-Ended Question

1. [If caregiver-completed] Is there anything additional that you would like to share about your child's experience living with a brother or sister who has congenital CMV?
   [If sibling/self-completed] Is there anything additional that you would like to share about living with a brother or sister who has congenital CMV?

   ☐ Yes [provide space for open-ended response]

   ☐ No

Demographics

[If caregiver-completed] This next part of the questionnaire will ask questions about the sibling.

[If sibling/self-completed] This next part of the questionnaire will ask questions about you.

2. [If caregiver-completed] What gender identity best describes the sibling?
   [If sibling/self-completed] What gender identity best describes you?

   ☐ Female

   ☐ Male

   ☐ Gender fluid/nonbinary/a gender identity not listed

   ☐ Prefer not to answer

3. If caregiver-completed] What race(s) or ethnicity(ies) do you consider the sibling to be? Please select all that apply.
   [If sibling/self-completed] What race(s) or ethnicity(ies) do you consider yourself to be? (Please select all that apply.)

   [If CG S2 = United States]

   ☐ African American or Black

   ☐ Alaska Native, American Indian, or Native American

   ☐ Asian or Asian American

   ☐ Hispanic, Latina/Latino, Latine, or Latinx

   ☐ Middle Eastern and/or North African

   ☐ Native Hawaiian and/or Pacific Islander

   ☐ White

   ☐ A race or ethnicity not listed

   ☐ Prefer not to answer [Exclusive]

[If CG S2 = Canada]

- ☐ Arab
- ☐ Black
- ☐ Chinese
- ☐ Filipino
- ☐ Indigenous (for example, member of a First Nation, Inuit, or Métis organization)
- ☐ Korean
- ☐ Japanese
- ☐ Latin American
- ☐ South Asian (for example, East Indian, Pakistani, Sri Lankan)
- ☐ Southeast Asian (for example, Vietnamese, Cambodian, Laotian, Thai)
- ☐ West Asian (for example, Iranian, Afghan)
- ☐ White
- ☐ A race or ethnicity not listed
- ☐ Prefer not to answer [Exclusive]

4. [If caregiver-completed] What is the sibling's current grade in school?

- ☐ Not enrolled in school
- ☐ Preschool
- ☐ Pre-kindergarten
- ☐ Kindergarten
- ☐ 1
- ☐ 2
- ☐ 3
- ☐ 4
- ☐ 5
- ☐ 6

- ☐ 7
- ☐ 8
- ☐ 9
- ☐ 10
- ☐ 11
- ☐ 12
- ☐ Graduated high school

[If CG Q77 = 8-17 years] What is your current grade in school?

- ☐ I do not go to school
- ☐ 1
- ☐ 2
- ☐ 3
- ☐ 4
- ☐ 5
- ☐ 6
- ☐ 7
- ☐ 8
- ☐ 9
- ☐ 10
- ☐ 11
- ☐ 12
- ☐ Graduated high school

[If CG Q77 ≥ 18 years] What is the highest level of education you have completed?

- ☐ Less than high school

- ☐ Some high school
- ☐ High school diploma or equivalent (for example, GED)
- ☐ Some college but no degree
- ☐ Associate's degree (2-year college degree)
- ☐ College degree (for example, BA, BS)
- ☐ Some graduate school but no degree
- ☐ Graduate or professional degree (for example, MBA, MS, MD, PhD)
- ☐ Prefer not to answer

5. [If sibling/self-completed] **Please select the best response.**
   - ☐ I completed the questionnaire by myself
   - ☐ I completed the questionnaire with help from an adult
   - ☐ Someone else completed the questionnaire for me

Thank You Screen

Thank you for taking the time to complete this survey.
Please take note of the following number that can be used for future reference:

Please click Next to receive information about the incentive.

Incentive Landing Screen

As a token of our appreciation, your family will receive a $[CG S2 = US, insert 25; CG S2 = Canada, insert 34] e-gift card for completing this survey.

The email will be sent from Rybbon, an online e-gift card company, and will be sent to the email that was previously provided.

The subject of the email will be "Gift Card for Completion of Sibling Survey"; please check your spam folder.

You may close this screen.

Table S1. Overview of Health Utility Measures

Measure	Number of items	Administration	Recall period	Domains/attributes
**Caregiver survey**				
EQ-5D-5L [23]	5 items + EQ VAS	Caregiver completed	Today	5 domains:<br>▪ Mobility<br>▪ Self-care<br>▪ Usual activities<br>▪ Pain/discomfort<br>▪ Anxiety/depression
WPAI:SHP [20-22]	6 items	Caregiver completed	Past 7 days	4 domains:<br>▪ Absenteeism (work time missed)<br>▪ Presenteeism (impairment while working)<br>▪ Overall work performance

Measure	Number of items	Administration	Recall period	Domains/attributes
				▪ Nonwork activities
**Child survey**				
IQI [24]	7 items	Caregiver proxy reported: children aged 0-24 months[a]	Today	7 domains:<br>▪ Sleeping<br>▪ Feeding<br>▪ Breathing<br>▪ Stooling/poo<br>▪ Mood<br>▪ Skin<br>▪ Interaction
HuPS [25]	11 items	Caregiver proxy reported: children aged 2-4 years	Usual health	8 attributes:<br>▪ Vision<br>▪ Hearing<br>▪ Speech

Measure	Number of items	Administration	Recall period	Domains/attributes
				▪ Ambulation<br>▪ Dexterity<br>▪ Emotion<br>▪ Cognition<br>▪ Pain and discomfort
HUI [26,27]	15 items	Caregiver proxy reported: children aged 5-7 years and 8-17 years with cognitive disability	Usual health	HUI2 attributes:<br>▪ Sensation<br>▪ Mobility<br>▪ Emotion<br>▪ Cognition<br>▪ Self-Care<br>▪ Pain<br>▪ HUI3 attributes:<br>▪ Vision
	40 items	Child self-assessment via caregiver interview: children aged 8-11 years with no cognitive disability and aged 12-17 years with visual		

Measure	Number of items	Administration	Recall period	Domains/attributes
		disability and no cognitive disability		▪ Hearing<br>▪ Speech<br>▪ Ambulation<br>▪ Dexterity<br>▪ Emotion<br>▪ Cognition<br>▪ Pain
	15 items	Child self-assessment; children aged 12-17 years with no cognitive or visual disabilities		
**Sibling survey**				
EQ-5D-Y-3L [28]	5 items + EQ VAS	▪ Caregiver completed: siblings aged 4-7 years	Today	5 domains:<br>▪ Mobility<br>▪ Self-care<br>▪ Usual activities<br>▪ Pain/discomfort

Measure	Number of items	Administration	Recall period	Domains/attributes
		▪ Sibling self-completed: siblings aged 8-17 years		▪ Anxiety/depression
EQ-5D-5L [23]	5 items + EQ VAS	Sibling self-completed: aged ≤ 18 years	Today	5 domains: ▪ Mobility ▪ Self-care ▪ Usual activities ▪ Pain/discomfort ▪ Anxiety/depression

EQ VAS = EQ visual analogue scale; HUI = Health Utilities Index; HUI2 = Health Utilities Index Mark 2; HUI3 = Health Utilities Index Mark 3; HuPS = Health Utilities Preschool; IQI = Infant Health-Related Quality of Life Instrument; WPAI:SHP = Work Productivity and Activity Impairment Questionnaire: Specific Health Problem.

[a]The instrument is designed for infants aged 0 to 12 months. Because no other instrument is currently available to measure utility in children aged 13 to 24 months, the developer has granted permission to use the IQI in this study for children aged up to 24 months.

Table S2. Disease Severity at Birth

Survey question	Asymptomatic	Isolated hearing loss[a]	Mild[b]	Moderate to severe[c]
**Did the child have any of the following at birth?**				
Abnormal blood test results lasting < 2 weeks[d]			✓	
Abnormal blood test results lasting ≥ 2 weeks[d]				✓
Abnormal brain imaging[e]				✓
Enlarged liver			✓	
Enlarged spleen			✓	
Eye/vision problems			✓	
Feeding difficulties			✓	
Hearing loss		✓		
Jaundice (high bilirubin levels)			✓	
Low birthweight			✓	
Low muscle tone			✓	
Low platelets lasting < 2 weeks			✓	
Low platelets lasting ≥ 2 weeks				✓

Survey question	Asymptomatic	Isolated hearing loss[a]	Mild[b]	Moderate to severe[c]
Pinpoint rash (also known as petechiae)			✓	
Seizures				✓
Microcephaly				✓

cCMV = congenital cytomegalovirus.

[a]Defined by Rawlinson et al. (2017) as "No apparent abnormalities to suggest congenital cytomegalovirus disease, but sensorineural hearing loss (≥ 21 decibels)."

[b]Defined by Rawlinson et al. (2017) as "Might occur with one or two isolated manifestations of congenital cytomegalovirus infection that are mild and transient (e.g., mild hepatomegaly or a single measurement of low platelet count or raised levels of alanine aminotransferase). These might overlap with more severe manifestations. However, the difference is that they occur in isolation." Defined in this study as having 1 or 2 of the listed manifestations only. If more than 2 of those listed or other manifestations are present, patients will be categorized as moderately to severely symptomatic.

[c]Defined by Rawlinson et al. (2017) as "Multiple manifestations attributable to congenital cytomegalovirus infection: thrombocytopenia, petechiae, hepatomegaly, splenomegaly, intrauterine growth restriction, hepatitis (raised transaminases or bilirubin)" or "Central nervous system involvement such as microcephaly, radiographic abnormalities consistent with cytomegalovirus central nervous system disease (ventriculomegaly, intracerebral calcifications, periventricular echogenicity, cortical or cerebellar malformations), abnormal cerebrospinal fluid indices for age, chorioretinitis, sensorineural hearing loss, or the detection of cytomegalovirus DNA in cerebrospinal fluid."

[d]For example, anemia, low blood count, low white blood cells, high liver enzymes.

[e]For example, medical imaging showing problems such as calcifications/calcium spots, white matter changes/differences, too many small surface folds, cysts, area of bleeding/hemorrhage, brain smaller than expected, or other problems.

Table S3. Out-of-Pocket and Work- or Productivity-Related Costs for Caregivers and Employment Impacts for Caregivers Stratified by cCMV Symptom Severity at Birth

	Disease severity at birth			
	Asymptomatic, n = 8	Isolated hearing loss, n = 7	Mild, n = 13	Moderate to severe, n = 199
**Annual out-of-pocket expenses, mean (SD), USD**				
Total	$3,432 ($4,737)	$12,867 ($11,694)	$18,079 ($27,770)	$17,137 ($35,592)
Medications, hearing aids, medical devices, equipment, or special foods	$351 ($698)	$5,079 ($5,144)	$2,984 ($4,641)	$2,588 ($5,957)
Occupational therapy, physical therapy, feeding therapy, or speech and language therapy	$975 ($1,868)	$7,431 ($10,729)	$10,168 ($20,100)	$9,333 ($22,680)
Other interventional therapies for your child with cCMV[a]	$1,800 ($4,206)	$0 ($0)	$4,043 ($9,840)	$2,984 ($12,984)
Transportation and lodging	$306 ($434)	$314 ($219)	$885 ($1,358)	$1,322 ($2,628)
Personal care (including respite care) from a paid provider	$0 ($0)	$43 ($113)	$0 ($0)	$910 ($4,214)

	Disease severity at birth			
	Asymptomatic, n = 8	Isolated hearing loss, n = 7	Mild, n = 13	Moderate to severe, n = 199
**Annual work- or productivity-related costs, mean (SD), USD**				
Total	$52,274 ($55,129)	$42,843 ($51,005)	$44,557 ($39,773)	$69,409 ($62,974)
Caregiver and other family members hours taken off work[b]	$8,003 ($7,252)	$9,278 ($4,019)	$9,615 ($8,443)	$15,897 ($25,727)
Caregiver reduced normal working hours[c]	$1,924 ($5,442)	$3,471 ($6,372)	$6,208 ($11,425)	$8,132 ($15,863)
Caregiver stopped work[d]	$33,665 ($36,194)	$20,172 ($34,456)	$23,392 ($30,923)	$34,636 ($32,325)
Family member stopped work[d]	$8,682 ($24,556)	$9,922 ($26,251)	$5,343 ($19,263)	$10,745 ($24,259)
**Lifetime out-of-pocket home adaptation and specialized schooling costs, mean (SD), USD**				
Total	$26 ($71)	$5,744 ($15,107)	$14,685 ($34,602)	$4,214 ($11,539)
Modifications to home environment	$25 ($71)	$5,743 ($15,106)	$15 ($38)	$1,854 ($7,056)
Specialized preschool or daycare	$1 ($1)	$1 ($1)	$14,669 ($34,581)	$1,905 ($7,306)
Private school (kindergarten through high school)	$0 ($0)	$0 ($0)	$0 ($0)	$455 ($3,375)

	Disease severity at birth			
	Asymptomatic, n = 8	Isolated hearing loss, n = 7	Mild, n = 13	Moderate to severe, n = 199
**WPAI:SHP subscale scores[e]**				
Absenteeism				
n	5	7	7	126
Mean (SD), %	2.7 (5.1)	6.4 (7.6)	22.2 (34.9)	14.0 (23.3)
Presenteeism				
n	5	7	6	121
Mean (SD), %	14.0 (15.2)	15.7 (5.3)	38.3 (26.4)	29.3 (23.6)
Work productivity loss				
n	5	7	6	121
Mean (SD), %	15.9 (17.9)	21.1 (8.2)	43.1 (27.1)	34.7 (26.3)
Activity impairment				
n	8	7	13	198

	Disease severity at birth			
	Asymptomatic, n = 8	Isolated hearing loss, n = 7	Mild, n = 13	Moderate to severe, n = 199
Mean (SD), %	18.8 (21.0)	28.6 (27.3)	35.4 (21.1)	35.4 (26.2)

cCMV = congenital cytomegalovirus; SD = standard deviation; USD = United States dollar; WPAI:SHP = Work Productivity and Activity Impairment Questionnaire: Specific Health Problem.

Note: All costs are reported in 2024 USD and were obtained from rounding the analysis results to the nearest dollar.

[a]For example, horse, aquatic, or music therapies.

[b]Cost due to work loss within the year was calculated using the reported average weekly number of hours lost multiplied by the hourly rates according to caregiver age and by 52.14 weeks.

[c]For caregivers who reduced the number of hours and were currently employed, cost due to reduced working hours were calculated by multiplying the hours of work per week before reduction minus their current working hours times 52.14 weeks and their hourly rates according to caregiver age.

[d]Cost estimates assume that the caregiver/family member reporting that they had stopped work and had not worked in the prior year.

[e]WPAI:SHP scores are expressed as impairment percentages, with higher scores indicating greater impairment and reduced productivity.

Table S4. Caregiver, Child With cCMV, and Sibling Health-State Utilities Stratified by cCMV Symptom Severity at Birth

	Disease severity at birth			
	**Asymptomatic**	**Isolated hearing loss**	**Mild**	**Moderate to severe**
**Caregiver**				
EQ-5D-5L utility index score				
n	8	7	13	199
Mean (SD)	0.95 (0.04)	0.92 (0.09)	0.84 (0.17)	0.85 (0.14)
**Child with cCMV**				
**Caregiver proxy reported**				
Age < 2 years				
IQI				
n	1	3	4	45
Mean (SD)	0.98	0.92 (0.03)	0.87 (0.10)	0.85 (0.12)
Median	-	0.93	0.91	0.86
Range	-	0.89-0.94	0.72-0.94	0.50-1.00
Age 2-4 years				
HuPS				
n	1	1	5	41
Mean (SD)	0.87	0.73	0.65 (0.36)	0.37 (0.37)
Median	-	-	0.74	0.35
Range	-	-	0.03-1.00	-0.21-1.00

	Disease severity at birth			
	Asymptomatic	Isolated hearing loss	Mild	Moderate to severe
Age 5-7 years				
HUI2				
n	3	3	3	38
Mean (SD)	0.77 (0.20)	0.64 (0.36)	0.88 (0.14)	0.55 (0.31)
Median	0.88	0.83	0.9	0.58
Range	0.54-0.90	0.22-0.85	0.73-1.00	0.11-0.95
HUI3				
n	3	3	3	38
Mean (SD)	0.58 (0.35)	0.46 (0.60)	0.69 (0.34)	0.31 (0.45)
Median	0.64	0.78	0.73	0.33
Range	0.20-0.89	-0.23-0.83	0.33-1.00	-0.31-0.93
Age 8-17 years[a]				
HUI2				
n	1	0	1	32
Mean (SD)	0.40	-	0.76	0.38 (0.24)
Median	-	-	-	0.31
Range	-	-	-	0.06-0.87
HUI3				
n	1	0	1	32
Mean (SD)	0.18	-	0.22	0.02 (0.32)

	Disease severity at birth			
	Asymptomatic	Isolated hearing loss	Mild	Moderate to severe
Median	-	-	-	-0.06
Range	-	-	-	-0.29-0.87
**Caregiver administered, self-reported**				
Age 12-17 years				
HUI2				
N	0	0	0	2
Mean (SD)	-	-	-	0.38 (0.22)
Median	-	-	-	-
Range	-	-	-	0.22-0.54
HUI3				
N	0	0	0	2
Mean (SD)	-	-	-	0.03 (0.20)
Median	-	-	-	-
Range	-	-	-	-0.11-0.18
**Self-administered, self-reported**				
Age 12-17 years				
HUI2				
n	0	0	0	9
Mean (SD)	-	-	-	0.76 (0.13)
Median	-	-	-	0.76

	Disease severity at birth			
	Asymptomatic	Isolated hearing loss	Mild	Moderate to severe
Range	-	-	-	0.59-1.00
HUI3				
n	0	0	0	9
Mean (SD)	-	-	-	0.58 (0.21)
Median	-	-	-	0.47
Range	-	-	-	0.35-1.00
**Caregiver administered, self-reported**				
Age 8-11 years				
HUI2				
n	2	0	0	4
Mean (SD)	0.88 (0.00)	-	-	0.80 (0.24)
Median	0.88	-	-	0.87
Range	0.88-0.88	-	-	0.46-1.00
HUI3 (n = 7)				
n	2	0	0	5
Mean (SD)	0.88 (0.08)	-	-	0.67 (0.51)
Median	0.88	-	-	0.85
Range	0.82-0.93	-	-	-0.23-1.00
**Sibling**				
**Caregiver proxy reported**				

	Disease severity at birth			
	**Asymptomatic**	**Isolated hearing loss**	**Mild**	**Moderate to severe**
Age 4-7 years				
EQ-5D-Y-3L				
n	4	3	7	56
Mean (SD)	1.00 (0.00)	0.83 (0.05)	0.83 (0.14)	0.91 (0.15)
Median	1	0.85	0.86	0.99
Range	-	0.77-0.87	0.61-1.00	0.35-1.00
**Self-completed**				
Age 8-17 years				
EQ-5D-Y-3L				
n	4	1	2	64
Mean (SD)	0.89 (0.18)	1	0.98 (0.03)	0.91 (0.12)
Median	0.97	-	0.98	0.93
Range	0.63-1.00	-	0.96-1.00	0.29-1.00
Age ≥ 18 years				
EQ-5D-5L				
n	2	0	0	5
Mean (SD)	1.00 (0.00)	-	-	0.87 (0.24)
Median	1	-	-	1
Range	-	-	-	0.45-1.00

cCMV = congenital cytomegalovirus; HUI = Health Utilities Index; HUI2 = Health Utilities Index Mark 2; HUI3 = Health Utilities Index Mark 3; HuPS = Health Utilities Preschool; IQI = Infant Health-Related Quality of Life Instrument; SD = standard deviation.

[a]The HUI questionnaire was proxy reported by caregivers because of developmental delay or disability in these children.

Note: Utility index scores are based on a scale from 0 (death) to 1 (full health).
